# **Outcomes of the novel EXIME prostate stent: initial experience in a South African setting**

**DOI:** 10.1007/s00345-026-06439-5

**Published:** 2026-05-04

**Authors:** Daniel Da Silva Ferreira, Chris Christofides, Ahmed Adam

**Affiliations:** Department of Urology, Faculty of Health Sciences, University of the Witwatersrand, Johannesburg, Gauteng South Africa

**Keywords:** Benign Prostatic Hyperplasia, Sexual function, Minimally invasive, African setting, Urinary retention, Temporary prostatic stent, EXIME prostatic stent, Innovation, Innovative, Novel

## Abstract

**Purpose:**

Urinary retention due to Benign Prostatic Hyperplasia (BPH) is a frequent and serious urological emergency. This study evaluated the efficacy and clinical outcomes of the EXIME temporary prostatic stent as an alternative to traditional catheterisation.

**Methods:**

A prospective study including 50 male patients presenting with BPH-related urinary retention or those awaiting definitive surgery was performed. The EXIME stent was inserted as an outpatient for a one-month indwelling period. Primary endpoints included voiding efficacy and ease of use. Secondary outcomes assessed sexual function restoration, pain and bacterial colonisation rates.

**Results:**

Spontaneous voiding was achieved in all 50 patients with a median maximum flow rate (Qmax) of 17.8 ml/s [IQR 12.9–21.7] and a median post-void residual (PVR) of 20 ml [IQR 10–37]. A significant restoration of sexual activity was observed, with a resumption rate of 88% among previously sexually active men (*p* < 0.001). Surrogate markers for bacterial colonisation improved with dipstick nitrite positivity dropping from 26% pre-insertion to 0% at removal (*p* < 0.001). Procedurally, 90% of insertions were graded as extremely easy to easy (VAS 1–3) and 84% of patients reported only minimal to uncomfortable pain (VAS 1–3). The failure rate was 4% with only two early removals documented.

**Conclusion:**

This study represents the first clinical experience on this novel innovation on the continent of Africa, demonstrating a safe, effective, and well-tolerated alternative to traditional catheterization by providing immediate symptom relief and improved outcomes regarding sexual function and bacterial colonisation thus supporting its use in the management of BPH-related urinary retention.

## Introduction

Urinary retention represents one of the most frequent urological emergency presentations encountered within the University of The Witwatersrand circuit hospitals. This condition is commonly secondary to Benign Prostatic Hyperplasia (BPH) and is characterized by the incomplete emptying of the urinary bladder and necessitates urgent bladder decompression. Currently, the standard of care for immediate decompression involves the insertion of a transurethral or suprapubic Foley catheter. Whilst effective for drainage, the use of indwelling Foley catheters is associated with significant drawbacks. Clinically and anecdotally, patients managed with Foley catheters experience a significant decline in quality of life, frequent urinary tract infections, increased socioeconomic burden and negative impact on self-image [1, 2]. Given these challenges, there is a pressing need for alternatives that offer the same efficacy as traditional catheterisation but without the associated morbidity. This study seeks to prospectively investigate an alternative to the Foley catheter that has been used worldwide, the EXIME *(Rocamed*,* Signes*,* France)* temporary prostatic stent - a device that can be inserted with a simple lubricant at the patient’s bedside without the need of any complex instrumentation in the hope that this information will help us to improve the future care and comfort of urine drainage in our setting.

## Materials and methods

### Cohort, criteria, setting and sample size

The study was performed as a prospective observational study with voluntary participation requiring written informed consent. The study was conducted between August 2025 and January 2026 specifically within the Urology Outpatient Department (UOPD) and the Emergency Department at Charlotte Maxeke Johannesburg Academic Hospital (CMJAH) based in Johannesburg, South Africa. The study population consisted of black male patients aged 40 years and older with clinically or histologically diagnosed Benign Prostatic Hyperplasia (BPH) presenting with either acute or chronic urinary retention, as well as those with an indwelling transurethral or suprapubic Foley catheter awaiting definitive surgical management. This specific race demographic was not an inclusion criteria but rather reflects the catchment population of the public-sector tertiary referral hospital where the study was conducted. Patients with known or suspected prostate cancer, contraindication to lidocaine or urethral drainage, any degree of urinary incontinence, prostate volumes measuring more than 100 ml or bladder neck to prostatic apex distance of more than 7 cm measured on transabdominal ultrasonography, any known bleeding diatheses or concurrent anticoagulant therapy and the presence of any untreated urinary tract infection (UTI) were excluded. All patients in the cohort utilised a Foley catheter prior to EXIME stent insertion for varying amounts of time and had either previously failed a trial without catheter (TWOC) whilst receiving alpha-blocker therapy with either tamsulosin or doxazosin or were planned for upfront definitive surgery based on the presence of hydronephrosis with renal insufficiency. Other factors that may have prohibited medical management were not present in the cohort. After Foley catheter reinsertion, medical therapy was discontinued as per institutional protocol whilst awaiting definitive surgical management. No patients received BPH medication during the EXIME stent indwelling. Following stent removal, all patients were re-catheterised and prioritised for definitive surgery due to prolonged surgical waiting times at our institution.

A sample size of 50 patients provided sufficient statistical power assuming a success rate of 85% (based on efficacy rates of similar temporary stents in the literature [3, 4]) and a margin of error of 10% with a 95% confidence interval. Ethical clearance (Certificate No. M250323) for the study was granted by both the chief executive officer of CMJAH as well as the Wits Human Research Ethics Committee (HREC).

### Data collection instruments

Data collection and all functional assessments were conducted by a single investigator to ensure consistency in measurement technique and to eliminate inter-observer variability. Measurements were taken at the pre-insertion visit and again at the visit one month later. The data collection involved three distinct modalities:

1. Clinical Interview and File Review:

Demographic data, relevant medical history and inclusion/exclusion criteria verification was obtained through direct patient interview and review of the patients clinical file. Sexual activity was assessed qualitatively during the clinical interview by asking patients whether they were able to successfully engage in sexual intercourse (a binary ‘yes/no’ response) during each treatment phase without the use of a validated psychometric questionnaire.

2) Subjective Assessments (Patient and Clinician Reported):

To quantify subjective experiences, standardised scaling systems were utilised. A visual analogue scale (VAS) was used to assess the ease of insertion and removal of the device based on the clinician’s opinion: 0 (effortless) and 10 (very difficult/impossible) as well as device tolerance based on patient opinion 0 (no pain) and 10 (worst possible pain).

3) Objective Clinical Measurements.

Functional outcomes were measured using standard urological equipment. Voiding function was assessed using uroflowmetry to obtain peak urinary flow rates (Qmax) and post-void residual volume (PVR) was assessed using trans-abdominal ultrasonography. Urinalysis was performed using urine dipstick testing before insertion and removal of the device. All assessments made use of standardised equipment to ensure reproducibility across the cohort.

Primary endpoints included voiding efficacy (Qmax, PVR) and ease of use (VAS). Secondary outcomes assessed sexual function restoration, pain (VAS) and bacterial colonisation rates via dipstick urinalysis.

### Device and Procedure

**Fig. 1:**
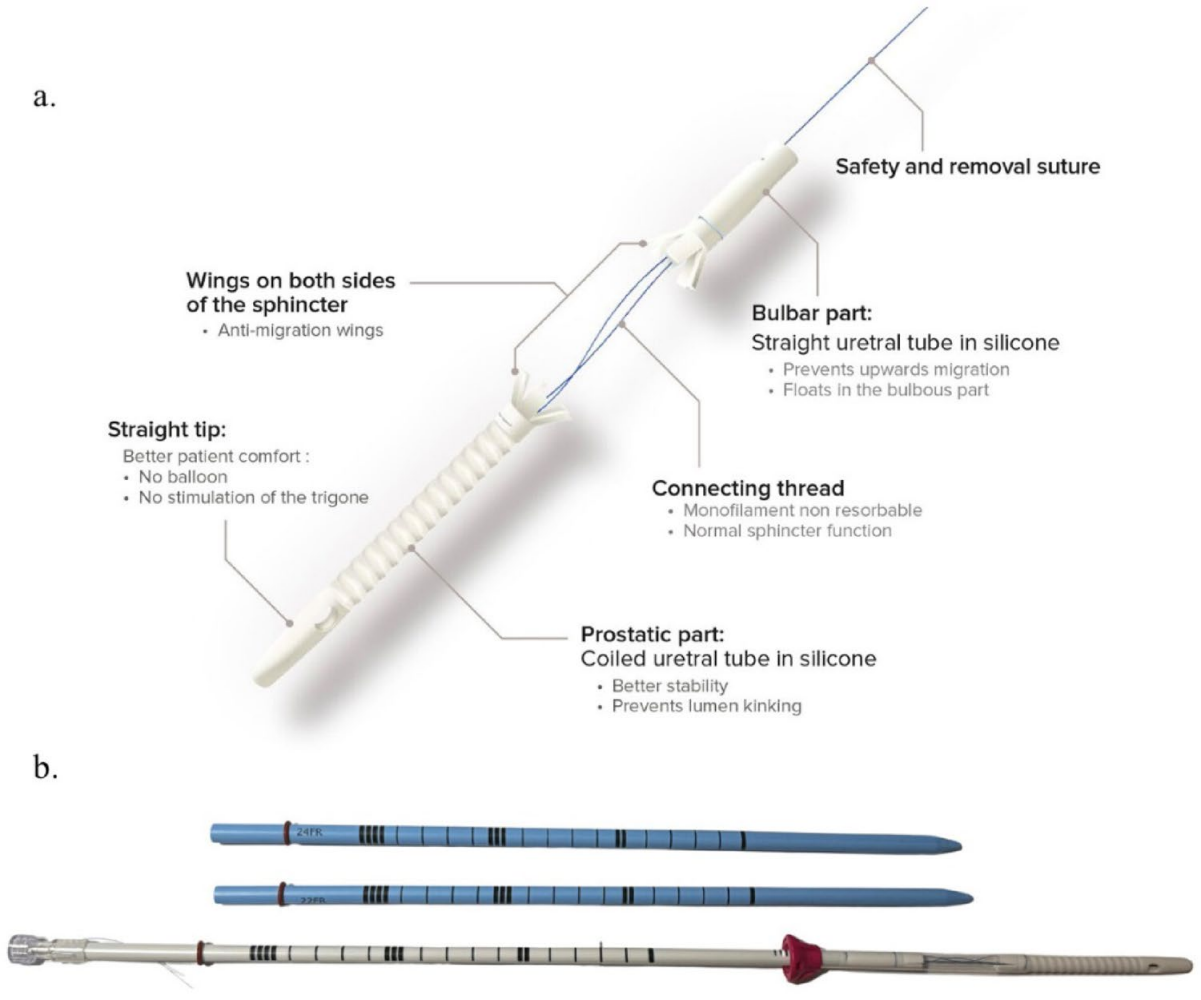
**a** The EXIME prostatic stent components and **b** urethral calibrators

The EXIME temporary prostatic stent is a device produced from silicone that utilises two ‘arms’ connected by a non-absorbable monofilament suture traversing the urinary sphincter to maintain patency in the prostatic and bulbar urethra whilst preserving urinary continence. Anti-migration winglets prevent the stent from unwanted expulsion during urination. Monofilament strings are attached to the distal end of the stent and protrude via the urethral meatus to facilitate removal of the device. The stent measures 80 mm in total length with a diameter of 20 Fr (Fig. 1a *– components of the EXIME stent)*.

All patients were required to have adequately filled bladders of > 200 ml, either passively or actively filled via a Foley catheter prior to insertion. The device is inserted at the patient’s bedside using a lubricating local anaesthetic (lidocaine) containing gel after sequential dilatation with urethral calibrators to 22Fr and 24Fr respectively (as provided within the device packaging – Fig. 1b). Device positioning and PVR was confirmed on transabdominal ultrasound. Device removal was performed at the bedside one month after insertion either via traction on the distal strings using a forceps or endoscopically in theatre under local anaesthesia if bedside removal was unsuccessful. All patients in this study received a single dose of oral Ciprofloxacin 500 mg upon device insertion.

### Statistical analysis

Descriptive statistics were performed for all available measured variables. Categorial variables were expressed as frequencies and percentages whilst continuous variables were represented as median values with an interquartile range (IQR). Comparative data was analysed with non-parametric tests via Pearson or Fishers exact Chi-square test. Statistical analysis was performed using IBM SPSS Statistics (version 29; IBM Corp., Armonk, NY). A significance level of *P* < 0.05 was set for this study.

## Results

**Fig. 2:**
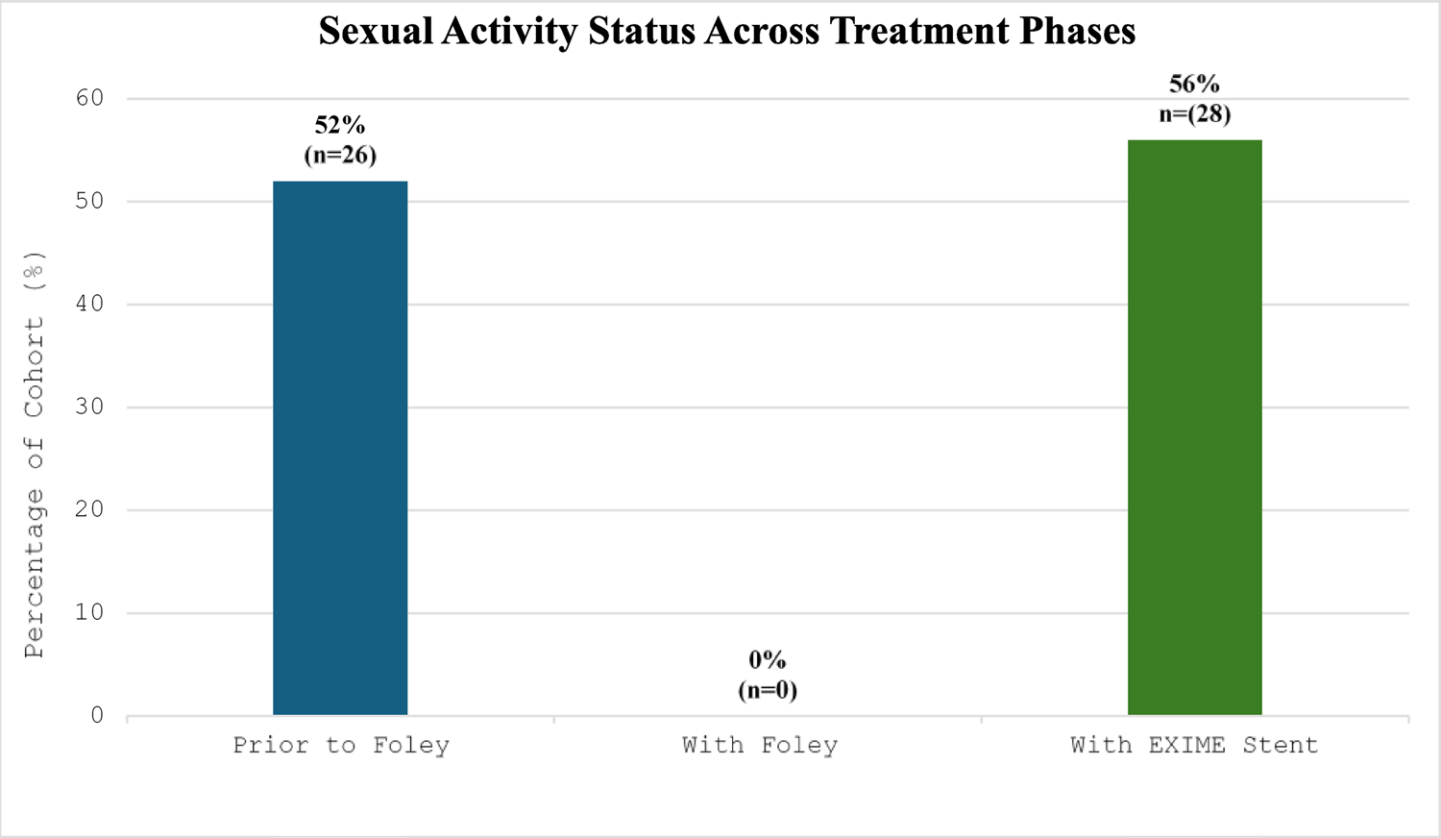
Bar graph highlighting sexual activity across treatment phases

**Fig. 3:**
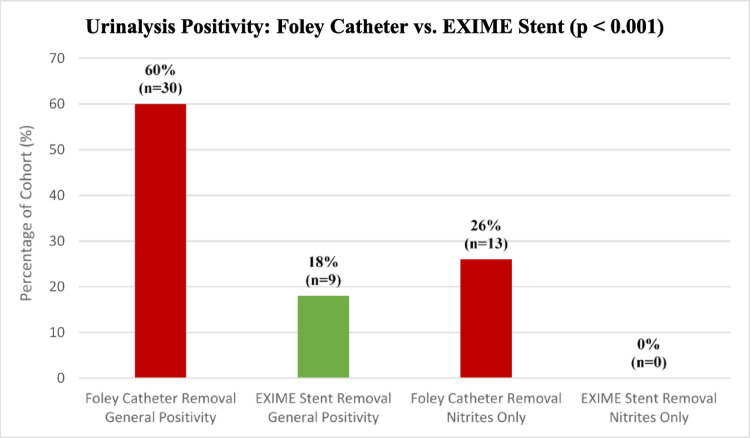
Comparison of urine dipstick positivity

Baseline demographics and clinical characteristics for the cohort (*n* = 50) have been summarised in Table 1. At the insertion visit, 52% (*n* = 26) of the cohort reported engaging in sexual intercourse prior to Foley catheter insertion and 100% (*n* = 50) of the cohort denied any sexual activity with a Foley catheter in situ. However, 56% (*n* = 28) of the cohort reported engaging in sexual activity with the EXIME stent in situ with approximately 88% (*n* = 23) of patients who were sexually active prior to Foley catheter insertion able to resume sexual function – this represents a statistically significant finding (*p* < 0.001, Fig. 2). In our department, latex Foley catheters are the standard of treatment, being changed at 6 week intervals. At the time of removal 60% (*n* = 30) of the cohort were found to have varying degrees of leukocyte esterase (LE) or nitrite reaction positivity on dipstick urinalysis - more specifically 26% (*n* = 13) of the cohort had some degree of nitrite reaction positivity however all patients were noted to be asymptomatic for UTI. Upon retrieval of the EXIME stent after 1 month, only 18% (*n* = 9) of the cohort were found to have urine dipstick positivity for varying degrees of LE and no patients (0%) were found to have any degree of nitrite reaction positivity - thus a significant inverse relationship between the Foley catheter and EXIME stent was noted with regard to urine dipstick positivity (*p* < 0.001, Fig. 3). There were no cases (0%) of symptomatic UTI requiring antibiotic therapy documented during the one-month indwelling period. All patients in the study were able to void spontaneously post stent insertion with the remainder of the voiding parameters summarised in Table 1. Ease of insertion and removal of the device was assessed based on a single physicians opinion using a VAS. 90% (*n* = 45) of the device insertions were graded as extremely easy, very easy or easy (*n* = 22, *n* = 16, *n* = 7) with 8% (*n* = 4) and 2% (*n* = 1) graded as moderate and somewhat difficult respectively. With regards to retrieval of the device, 95.9% were graded as extremely easy, very easy or easy (*n* = 23, *n* = 21, *n* = 3) and 2% (*n* = 1) as somewhat difficult. 1 case was described as extremely difficult (2%; *n* = 1). When assessing the patients experience of pain on insertion and removal of the device, a VAS scale was utilized. 84% (*n* = 42) of the cohort described the insertion as either minimal (level 1), mild (level 2) or uncomfortable pain (level 3) (*n* = 12, *n* = 18, *n* = 12), 14% (*n* = 7) experienced moderate pain (level 4) whilst one patient (2%) reported distressing pain (level 5). When assessing patients tolerance of retrieval, 87.8% (*n* = 43) of patients reported either minimal, mild or uncomfortable pain, 10% (*n* = 5) and 2% (*n* = 1) of patients reported moderate or distracting (level 5) pain respectively. 4% of the patient cohort (*n* = 2) did not complete the 1 month study period for reasons mentioned below. Device-related complications were graded according to the modified Manufacturer and User Facility Device Experience (MAUDE) classification system. Level I complications included transient rose-coloured urine (*n* = 8, 16%), transient urinary incontinence (*n* = 5, 10%) and retrograde ejaculation (*n* = 2, 4%), all resolved spontaneously within 48 h not requiring intervention. Level II complications occurred in 4% of the cohort (*n* = 2), consisting of one early stent removal on day 4 post insertion due to severe pain and one spontaneous stent expulsion on day 3 post insertion, both requiring minor clinical management. There was one (2%) Level III complication involving a snapped retrieval suture that necessitated endoscopic retrieval under local anaesthesia.

**Table 1:** Patient Demographics, Baseline Characteristics and Functional Outcomes (*n* = 50)

Parameter	Value	*p*-value
Baseline Demographics & Clinical History		
Age (years), Median [IQR]	66 [61–69]	-
Prostate Volume (ml), Median [IQR]	63 [40–78]	-
Duration of Foley Indwelling (months), Median [IQR]	4 [1–7]	-
Urine Retention Volume (Prior to Foley), Median [IQR]	301 [226–412]	-
Index Presentation, n (%)		
Chronic Urinary Retention	45 (90%)	-
Transurethral Foley Catheter	42 (84%)	-
Suprapubic Foley Catheter	3 (6%)	-
Acute Urinary Retention (Transurethral Foley)	5 (10%)	-
Anatomy, n (%)		
Median Lobe Enlargement (IPP present)	20 (40%)	-
Hydroureteronephrosis (at index presentation)	7 (14%)	-
Post-Insertion Voiding Parameters		
Spontaneous Voiding Rate, n (%)	50 (100%)	-
Qmax (ml/s), Median [IQR]	17.8 [12.9–21.7]	-
Voided Volume (ml), Median [IQR]	211.5 [162–288]	-
Post-Void Residual (ml), Median [IQR]	20 [10–37]	-
Comparative Functional Outcomes		
Sexual Activity, n (%)		
Pre-Foley Catheter Insertion	26 (52%)	-
With Foley Catheter In Situ	0 (0%)	-
With EXIME Stent In Situ	28 (56%)	-
Regain of sexual function (if active pre-Foley)	23/26 (88%)	< 0.001
Urinalysis (Dipstick Positivity), n (%)		
Combined Leukocyte Esterase (LE) or Nitrite Positive		
At Foley Removal (Baseline)	30 (60%)	-
At Stent Retrieval (1 Month)	9 (18%)	< 0.001
Nitrite Reaction Positive Only		
At Foley Removal (Baseline)	13 (26%)	-
At Stent Retrieval (1 Month)	0 (0%)	< 0.001

*IQR* Interquartile Range; *IPP * Intravesical Prostatic Protrusion; *Qmax * Maximum flow rate

## Discussion

Temporary prostatic stents represent a significant development in the management of BPH by providing a minimally invasive alternative to current Foley catheters, aiming to improve patient quality of life by alleviating urinary symptoms without the detrimental effect on sexual function, socioeconomic status and perceived self-image [1, 2]. The principal finding of this prospective study demonstrates that the EXIME temporary prostatic stent represents a safe, effective and feasible alternative to indwelling Foley catheterization for men with BPH-related urinary retention and aligns with the growing body of evidence suggesting that temporary prostatic stenting can bridge the gap between retention and definitive surgery without the “social and physical castration” often imposed by indwelling catheters [5, 6].

Prostatic stents emerged in the 1980 s to provide immediate relief of urinary obstruction by physically dilating the prostatic urethra. The designs ranged from metal (*Porges Urospiral by Coloplast*,* Allium by Allium Medical)* or silicone (*Coreflow by Prostalund*,* Spanner by SRS medical*) stents to thermo-expansible devices *(Memokath and Memotherm by Bard)* which have evolved to improve biocompatibility and functionality [4, 7]. They have proven particularly useful in patients who are poor surgical candidates or those who require temporary relief before undergoing definitive treatments.

Prostatic stents have been found to significantly reduce International Prostate Symptom Score (IPSS) and improve Qmax in the short term [8], furthermore, a multicenter trial by McNicholas et al. reported high patient satisfaction rates and low incidence of adverse events suggesting it as a viable alternative to traditional catheterization [9]. The functional outcomes observed in our cohort are comparable to those reported in recent international literature. Our observed success rate of 100% for spontaneous voiding is consistent with the high technical success rates reported by Baboudjian et al. in their evaluation of the EXIME stent [7]. Our median Qmax of 17.8 ml/s [IQR 12.9–21.7] compares favourably to other temporary stent devices currently on the market. For instance, studies on *The Spanner* stent have reported mean flow rates of approximately 10–12 ml/s in similar retention cohorts [10, 11] while earlier generations of thermo-expandable stents like the *Memokath* typically achieved flow rates of 11–15 ml/s [12]. Crucially, the device failure rate in our study was low (4%) compared to historical stent data. Previous iterations of prostatic stents were often plagued by migration and obstruction with rates ranging from 15% to 40% [10, 13]. The low migration rate observed here suggests that the EXIME’s specific design features - likely the distal anchor and anti-migration wings, effectively mitigate the expulsion risks that limited the widespread adoption of previous devices [7, 14].

The high ease-of-use scores and low pain scores in our study corroborate the device’s feasibility in a bedside setting. Unlike early experiences with other stents that often required endoscopic removal by design [13], our study required endoscopic intervention in only one case - validating the safety of the retrieval tether system for outpatient management. This ease of use is critical for resource constrained settings where minimizing operating theatre usage is a priority.

The Foley catheter is by far the most common modality used in clinical practice for urinary drainage in cases of urine retention [15]. Bacterial contamination is systematically observed after 5 days of catheterization and thus some catheters are coated with specific antimicrobial agents to prevent or delay infection however their price is much higher than that of the standard catheter without coating [16]. The drop in nitrite positivity from 26% (pre-insertion) to 0% (at removal) in our study represents a stark improvement over the natural history of standard catheterization. It is well documented that daily bacteriuria risk increases by 3–8% per day with an indwelling catheter, reaching nearly 100% at 30 days due to biofilm formation on the catheter surface [17]. While the administration of prophylactic ciprofloxacin at insertion in our protocol is a major confounding factor, the maintenance of nitrite negativity suggests that the EXIME stent allows for the natural washout mechanisms of spontaneous voiding, preventing the stasis and ascending colonization typical of static Foley catheters [18]. However, as urine cultures were not performed, these dipstick findings must be interpreted strictly as a reduction in surrogate markers for bacteriuria rather than a confirmed reduction in clinical infection risk. These results must be interpreted as hypothesis-generating and definitive conclusions regarding infection prevention cannot be made without simultaneous culture data.

Maneuvers of disconnection and reconnection of the catheter to the tubing of the collecting bag [19], diagnosis of urinary infection by urine analysis with evaluation of the microbial sensitivity to antibiotics and treatment of urinary infection increases the cost of catheterisation compounded by the fact that catheterization remains the leading cause of nosocomial infection in hospitals and contributes significantly to the worsening of antibiotic resistance [16, 20].

Sexual function is severely hampered by the presence of a transurethral catheter and although it is possible to engage in coitus, the negative effects on body image, lack of sexual self-esteem and possible pain all contribute to poor sexual function. Our study showed that sexual intercourse is not only possible but also safe with the EXIME stent in situ. Whilst the deleterious impact of Foley catheters on sexual function is well-established with qualitative studies describing the “stigmatizing” and hindering nature of external appliances [21, 22], there is a paucity of data quantifying the restorative potential of temporary stents. Our finding that 88% of priorly sexually active men resumed sexual activity significantly contributes to this under-researched area. Unlike indwelling catheters which physically impede erection and intercourse, the EXIME stent resides entirely within the urethra thus preserving the anatomical integrity required for sexual activity. Although we observed a 7.1% rate of retrograde ejaculation - likely due to the stent holding the bladder neck open, this trade-off is arguably negligible compared to the complete cessation of sexual activity necessitated by a Foley catheter. This finding underscores the device’s role not just as a drainage tool but as a holistic quality-of-life intervention.

Our results must be interpreted within the context of certain limitations. First, this was a single-centre observational study with a modest sample size (*n* = 50) potentially limiting the transferability of the findings. It is important to recognize that the high success and low complication rates observed may be partially attributed to our strict selection criteria and cohort demographic. In a more heterogeneous population including those with larger prostates or significant co-morbidities, the outcomes may differ and thus must be interpreted within our specific setting. Second, the lack of a randomized control arm means that comparisons to Foley catheter outcomes are based on intra-individual changes (patients serving as their own controls) and global data rather than a parallel control group. Third, the assessment of sexual outcomes was based on binary patient self-reporting during clinical interviews rather than a validated, multi-domain questionnaire like the International Index of Erectile Function (IIEF). This approach may oversimplify the complexities of sexual function and limits our ability to precisely quantify changes sexual function. Additionally, the follow-up period was limited to one month, while sufficient for a “bridge to surgery” indication, long-term durability and encrustation risks beyond 30 days remain unassessed in this protocol.

Temporary prostatic stents represent a valuable option in the armamentarium for managing BPH. In this cohort, the functional and quality-of-life outcomes observed with the EXIME temporary prostatic stent appeared favourable when compared to the patients’ baseline experiences with Foley catheterization. However, as these observations are based on intra-individual changes and historical benchmarks rather than a concurrent control group, these findings should be considered preliminary. By allowing patients to void naturally and maintain sexual function while awaiting definitive surgery, it addresses the major psychological and physical drawbacks of the current standard of care. Future multi-centre randomized trials are warranted to confirm these benefits and establish this modality as a routine alternative for temporary bladder drainage.

## Data Availability

The data that support the findings presented in this study are available from the corresponding author upon reasonable request.
